# Effect of Intermittent Fasting on Non-Alcoholic Fatty Liver Disease: Systematic Review and Meta-Analysis

**DOI:** 10.3389/fnut.2021.709683

**Published:** 2021-07-12

**Authors:** Cong Yin, Zihan Li, Yulin Xiang, Hongbing Peng, Ping Yang, Shijun Yuan, Xueyan Zhang, You Wu, Min Huang, Juan Li

**Affiliations:** ^1^Hubei Province Key Laboratory of Traditional Chinese Medicine Resource and Chemistry, Hubei University of Chinese Medicine, Wuhan, China; ^2^College of Basic Medical Sciences, Hubei University of Chinese Medicine, Wuhan, China

**Keywords:** fasting, intermittent fasting, time restricted feeding, non-alcoholic fatty liver disease, systematic review, meta-analysis

## Abstract

**Background:** Weight loss by lifestyle modification is the cornerstone therapy of non-alcoholic fatty liver disease (NAFLD). Intermittent fasting has shown favorable effects on body weight (BW) and relevant indicators of NAFLD in several reports.

**Objective:** To estimate the effects of intermittent fasting on adults with NAFLD.

**Materials and methods:** Literature searches were conducted on PubMed, EMBASE, Web of Science, Cochrane Library, and ClinicalTrials.gov from inception to May 10, 2021.

**Results:** A total of six studies involving 417 patients with NAFLD were included. In the meta-analysis, there were significant differences in BW, body mass index (BMI), alanine aminotransferase (ALT), and aspartate transaminase (AST) between the control and fasting group. Up to now, there is no significant difference in triglycerides (TG), total cholesterol (TC), and other metabolic parameters between the two groups.

**Conclusions:** Intermittent fasting is beneficial for weight management and liver enzyme improvement, but long-term feasibility and safety of intermittent fasting should be conducted in further studies.

## Introduction

Non-alcoholic fatty liver disease (NAFLD) is the predominant cause of chronic liver diseases worldwide, causing considerable liver-related and extrahepatic morbidity and mortality (1). It is a clinical and pathological syndrome characterized by excessive fat deposits in hepatocytes, resulting from causes other than factors including alcohol and liver damage (2). Patients with NAFLD have an increased risk of cardiovascular disease, type 2 diabetes, and even chronic kidney disease, and mortality from liver-related and non-liver-related causes appears more significant than in the general population (3–6). The increasing prevalence of the disease worldwide is thus particularly worrisome. Given that there are currently no drugs approved for the treatment of NAFLD, lifestyle modification (through dietary and exercise interventions) remains the underlying approach for the treatment of NAFLD (7). Paired liver biopsy studies have demonstrated that a ≥5% loss of body weight (BW) is associated with significant reductions in hepatic steatosis (HS), ≥7% loss of weight is associated with a decrease in hepatic inflammation, and a ≥10% loss is associated with a reduction in fibrosis (8).

Since dietary interventions play an important role in weight loss management, it is necessary to study suitable dietary interventions for patients with NAFLD. Intermittent fasting, which includes alternate-day fasting, and other forms of periodic caloric restriction have already received attention from animal research scientists (9, 10). It has been shown that fasting may benefit weight management and improve cardiovascular and metabolic risks (11).

Although there exist some clinical trials assessing the effect of intermittent fasting on NAFLD, there has been no meta-analysis of these clinical trials, and there is a lack of evidence-based medical evidence on the effect of intermittent fasting on NAFLD (12–17). Therefore, in this study, we performed a systematic review and meta-analysis to include clinical trials that adopt fasting intervention for NAFLD and to evaluate the effect of fasting on factors associated with NAFLD.

## Materials and Methods

This research study was performed according to the guidelines of the preferred reporting items for systematic reviews and meta-analyses (PRISMA) (Supplementary Table 1) (18).

### Search Strategy

We searched PubMed, EMBASE, Cochrane Library, Web of Science, and ClinicalTrials.gov databases to find relevant studies up to May 10, 2021, in all languages using a combination of the main search terms “fasting” and “NAFLD.” The search strategy was designed to be as broad as possible. Then, the reference lists of the relevant studies were checked for additional studies. The detailed search strategy is presented in Supplementary Table 2.

Two authors (YC and LJ) independently reviewed the titles and abstracts. Discrepancies were resolved through discussions among the two authors. The two authors then independently analyzed the full text of the remaining studies to determine the final inclusion.

### Study Selection and Data Extraction

For this meta-analysis, inclusion criteria were as follows: (1) participants with NAFLD were adult humans aged ≥18 years across different countries; (2) the intervention group underwent fasting intervention alone or in conjunction with other lifestyle interventions; (3) the comparator group underwent no nutritional intervention (“control”) or a different type of nutritional intervention; (4) the full text was available and was written in English; (5) the trial included evaluation of at least one of the following outcomes: BW, body mass index (BMI), waist circumference (WC), fasting blood sugar/glucose (FBS/GLU), homeostasis model assessment of insulin resistance (HOMA-IR), fasting insulin (FINS), alanine aminotransferase (ALT), aspartate transaminase (AST), liver stiffness, total cholesterol (TC), triglycerides (TG), high-density lipoprotein cholesterol (HDL-C), or low-density lipoprotein cholesterol (LDL-C); and (6) the trial reported the mean value of changes from baseline (or values of post-intervention when unavailable) with SD (or suitable data to determine the parameter: SE or 95% CI). Animal studies and uncontrolled and cross-sectional studies were not included.

Two authors (YC and LJ) extracted data from the selected trials. This process was verified by another investigator (HM). An Excel database was used to extract the following information from the included studies: (1) study characteristics (e.g., names of the authors, year, sample size, and ages of the patients), (2) treatments, (3) methodological aspects, and (4) clinical outcomes. To obtain the data not presented in the original studies, we sent two e-mails 1 week apart to the corresponding authors.

### Risk of Bias Assessment

We followed the guidelines in the Cochrane Handbook for Systematic Reviews of Interventions to assess the risk of bias of these included studies, which included detection bias (outcome assessment blinding), selection bias (random sequence generation and allocation concealment), attrition bias (incomplete outcome data), and reporting bias (selective reporting) (19). We assessed every domain of bias in terms of methodological quality and assigned a rating of low or high risk of bias. If there was insufficient information to make an adequate assessment, the domain was evaluated as “unclear”.

### Statistical Analysis

A series of calculations were executed to standardize the data in terms of SD as some studies reported CIs. The conversion of 95% CI to SD was calculated by $SD=\sqrt{N}\times \frac{upper\;limit-\;lower\;limit}{2t};t=tinv\left(1-0.95,\;NE+NC-2\right)\;$ (19). The mean change from baseline to after placebo or treatment was calculated by $\bar{X_{c}}=\bar{X_{a}}-\bar{X_{b}}$, and the SD of change was calculated by $S_{c}=\sqrt{S_{a}^{2}{+S}_{b}^{2}-2\times corr\times S_{a}\times S_{b}}$ (19). We calculated SD for the change values by selecting 0.5 as the correlation coefficient (*r* = 0.5), and to make sure that the meta-analysis was not sensitive to the selected correlation coefficient, all analyses were repeated using correlation coefficients of 0.2 and 0.8.

The mean difference (MD) and its corresponding 95% CIs were calculated using the random effects model, which takes the between-study heterogeneity into account (19). Heterogeneity was assessed statistically using the standard chi-square *I*^2^ test. A random effects model using the double-arcsine transformation approach was used. *I*^2^ values ≤ 25, 25–50, 50–75, and >75% indicated no, small, moderate, and significant heterogeneities, respectively. To examine the potential sources of between-study heterogeneity, subgroup analyses were performed according to the type of control diet (religious intermittent fasting/modern intermittent fasting regimens). A sensitivity analysis was conducted only when three or more studies were included in the comparison. Sensitivity analysis was used to assess the robustness of the results of meta-analyses by sequentially removing individually included studies. The presence of the publication bias was checked for each outcome through funnel plots. Publication bias was assessed using the Egger regression asymmetry test when the comparison contained at least 10 studies. All statistical analyses were performed using STATA MP, version 16, and two-sided *p*-values < 0.05 were considered statistically significant.

## Results

### Study Selection and Study Characteristics

**Figure 1:**
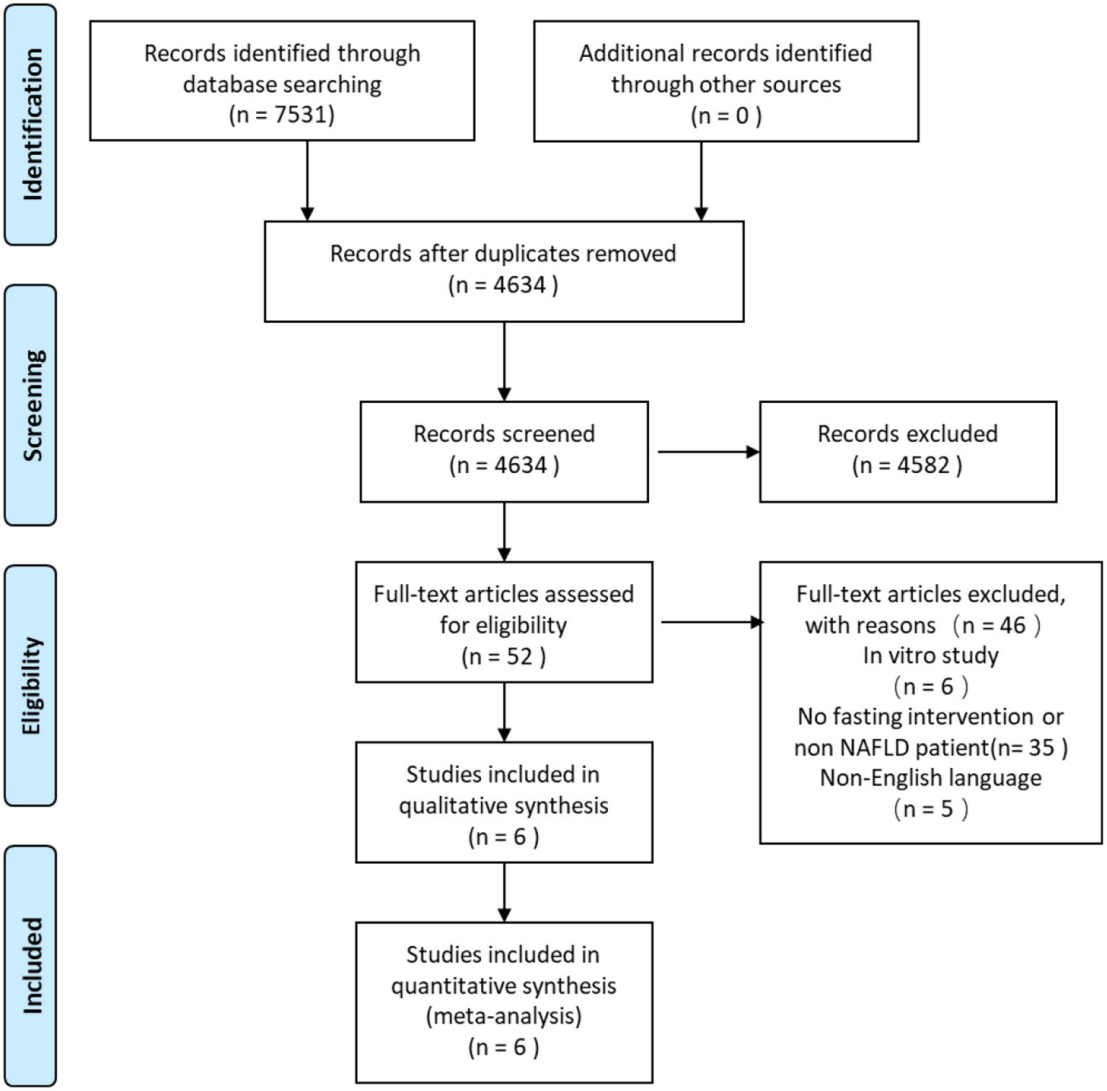
Preferred reporting items for systematic reviews and meta-analyses (PRISMA) flow diagram for screening and selection process of studies.

In the systematic search, a total of 7,531 citations were identified, and the study selection process is illustrated in Figure 1. After the removal of duplicates, 4,634 studies were assessed *via* their title and abstract for potential eligibility. In total, 4,582 studies were excluded, and 52 studies were retrieved for full-text analysis. Finally, six studies fulfilled the inclusion criteria of being a systematic review, and the characteristics of the included studies are summarized in Table 1.

**Table 1:** Cohort characteristics.

**References**	**Country**	**Design**	**Mode of intervention**	**Subjects**	**Sex**	**Age**		**Duration**	**Type**	**Outcomes**
				**T/C**	**M/F**	**T**	**C**			
Aliasghari et al. (12)	Iran	Comparative, double arm	Ramadan fasting, 15–16 h a day	42/41	57/26	37.59 (7.06)	35.80 (7.33)	30 d	Religious	BW, BMI, WC, FBS/GLU, HOMA-IR, FINS
			Non-fasting							
Cai et al. (13)	China	RCT	Time-restricted feeding, 16 h a day	95/79	29/66	35.52 (5.48)	34.54 (6.96)	12 week	Modern	BW, BMI, WC, FBS/GLU, TG, TC, LDL-C, HDL-C, liver stiffness
			Non-fasting							
Cai et al. (13)^*^	China	RCT	Alternate-day fasting, 22 h a day	90/79	35/60	35.52 (5.48)	34.54 (6.96)	12 week	Modern	BW, BMI, WC, FBS/GLU, TG, TC, LDL-C, HDL-C, liver stiffness
			Non-fasting							
Mari et al. (16)	Israel	Comparative, double arm	Ramadan fasting, 15–16 h a day	74/81	81/74	51.8 (20.9)	52.6 (19.3)	30 d	Religious	BMI, HOMA-IR, FINS, ALT, AST
			Non-fasting							
Rahimi et al. (17)	Iran	Comparative, double arm	Ramadan fasting, 15–16 h a day	34/26	39/21	46.03 (11.72)	49.58 (10.96)	30 d	Religious	BW, BMI
			Non-fasting							
Ebrahimi et al. (14)	Iran	Comparative, double arm	Ramadan fasting, 15–16 h a day	42/41	57/26	37.59 (7.06)	35.80 (7.33)	30 d	Religious	TG, TC, LDL-C, HDL-C
			Non-fasting							
Johari et al. (15)	Malaysia	RCT	Modified alternate-day calorie restriction, 18 h alternate-day	33/10	33/10	45.33 (10.77)	52.60 (12.03)	8 w	Modern	BW, BMI, ALT, AST, TG, TC, LDL-C, HDL-C
			Non-fasting							

*T, treatment; C, control; RCT, randomized controlled trial; M, male; F, female.*

* *This study has two fasting intervention groups*.

Seven datasets [one study (20) had two comparators: control and time-restricted feeding (TRF)] were included in this meta-analysis. The intervention group included 417 participants (range 30–95), and the comparator group included 278 participants (range 9–81). All studies did not distinguish between men and women. All five studies targeted the NAFLD population; only one study had non-alcoholic steatohepatitis (NASH) population in addition to the NAFLD population.

Two of the six studies were randomized controlled trials (RCTs), and on the specific methods of fasting, four used Ramadan fasting, two used alternate-day fasting, and one used TRF. On the days of fasting, the fasting time is almost always 15–16 h/day. Two studies restricted calories, and the other studies did not report caloric restriction. None of the studies cooperated with the exercise intervention. The duration of these researchers ranged from 4 to 12 weeks. BW was measured in all the six studies, BMI was reported in five studies, FBS/GLU, TG, TC, and HDL-C in four studies, WC and ALT in three studies, and AST, liver stiffness, FI, and HOMA-IR in two studies.

### Effect of Intermittent Fasting on BW

**Figure 2:**
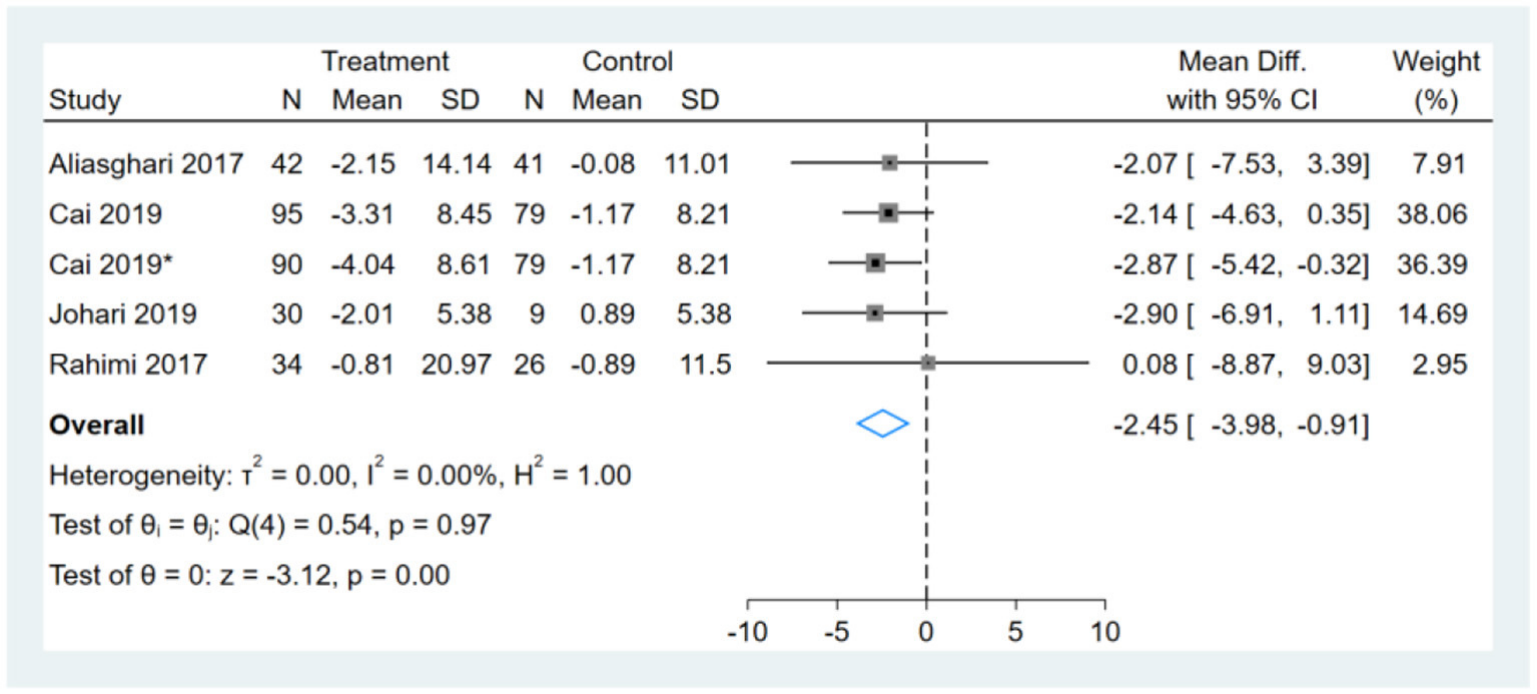
Forest plot analysis showing changes in body weight (BW). *This study has two fasting intervention groups.

One study had two comparators, and two datasets were included (13). Two studies used the same study population, and only one dataset was included (12, 14). One of the remaining three studies did not report BW (16). Therefore, a total of five BW datasets were included in the analysis. Meta-analysis shows that the effect of fasting on BW was statistically significant (MD, −2.45, 95% CI from −3.98 to −0.91, *p* ≤ 0.00) (Figure 2). Negative values favor fasting because the fasting group experienced more reduction in BW than did the control group.

### Effect of Intermittent Fasting on BMI

**Figure 3:**
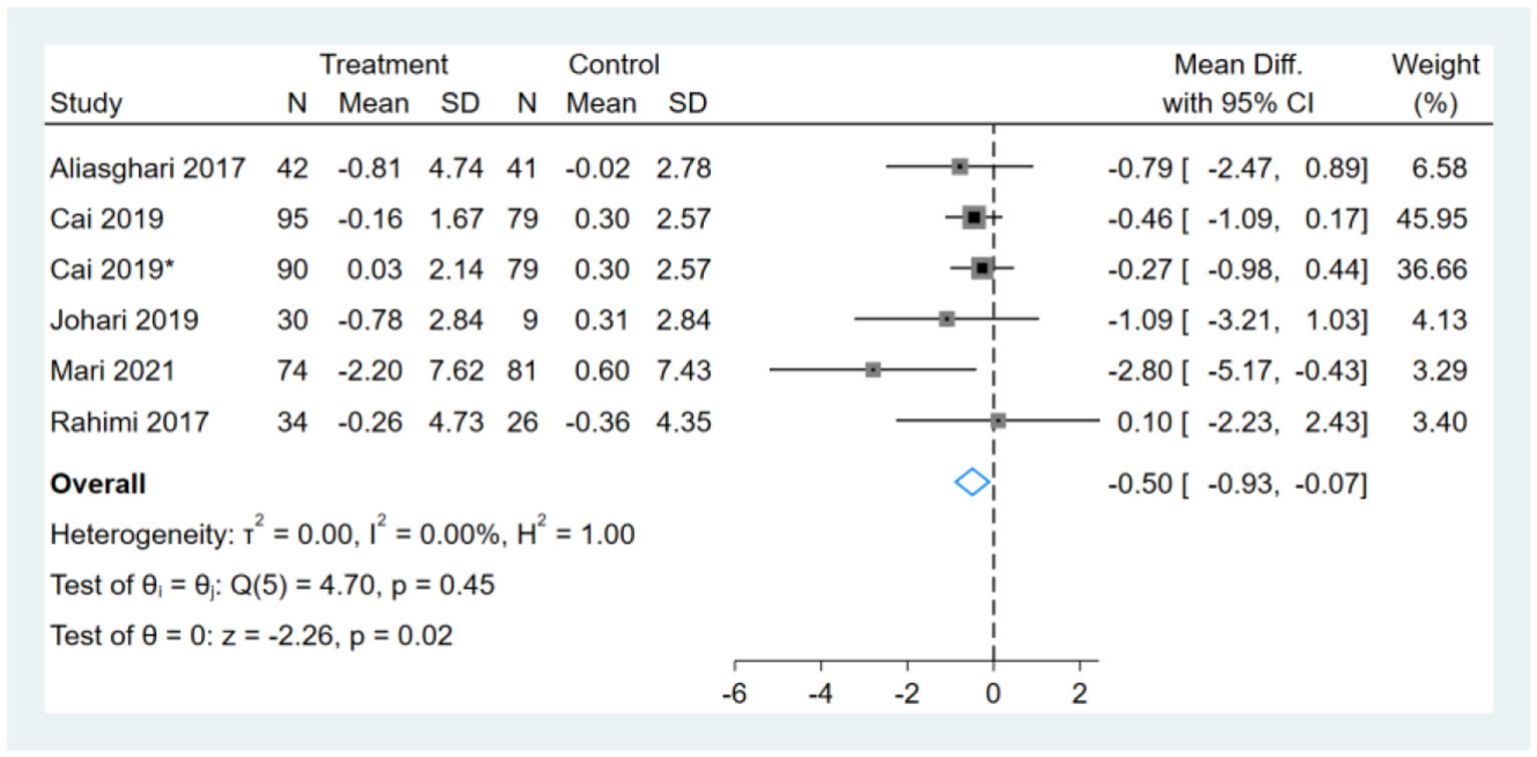
Forest plot analysis showing changes in body mass index (BMI). *This study has two fasting intervention groups.

One study had two comparators, and two datasets were included (13). Two studies used the same study population, and only one dataset was included (12, 14). Therefore, a total of six BMI datasets were included in the analysis. Meta-analysis shows that the effect of fasting on BMI was statistically significant (MD, −0.50, 95% CI from −0.93 to −0.07, *p* = 0.02) (Figure 3). Negative values favor fasting because the fasting group experienced more reduction in BMI than did the control group.

### Effect of Intermittent Fasting on WC

**Figure 4:**
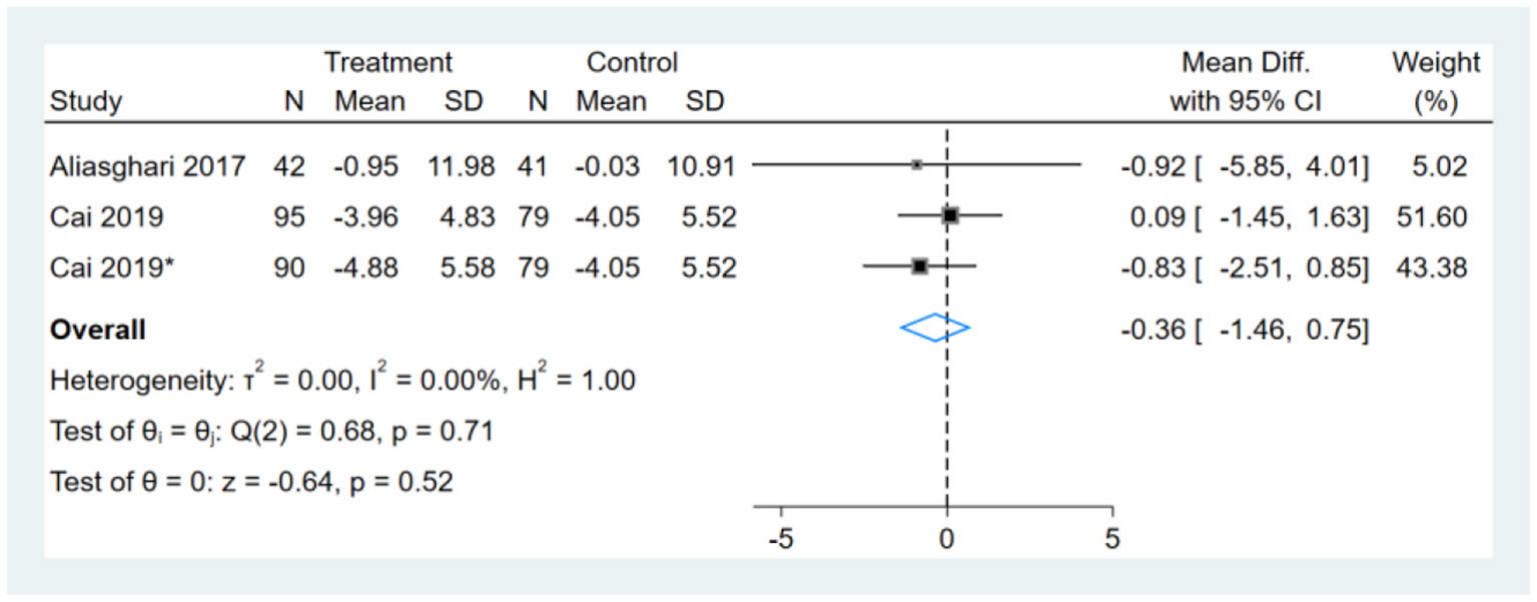
Forest plot analysis showing waist circumference (WC) changes. *This study has two fasting intervention groups.

Two studies reported WC results, one of them had two comparators, and two datasets were included (13). Therefore, a total of three WC datasets were included in the analysis. Meta-analysis shows that the effect of the fasting intervention was not significant for WC (Figure 4).

### Effect of Intermittent Fasting on Serum FBS/GLU

**Figure 5:**
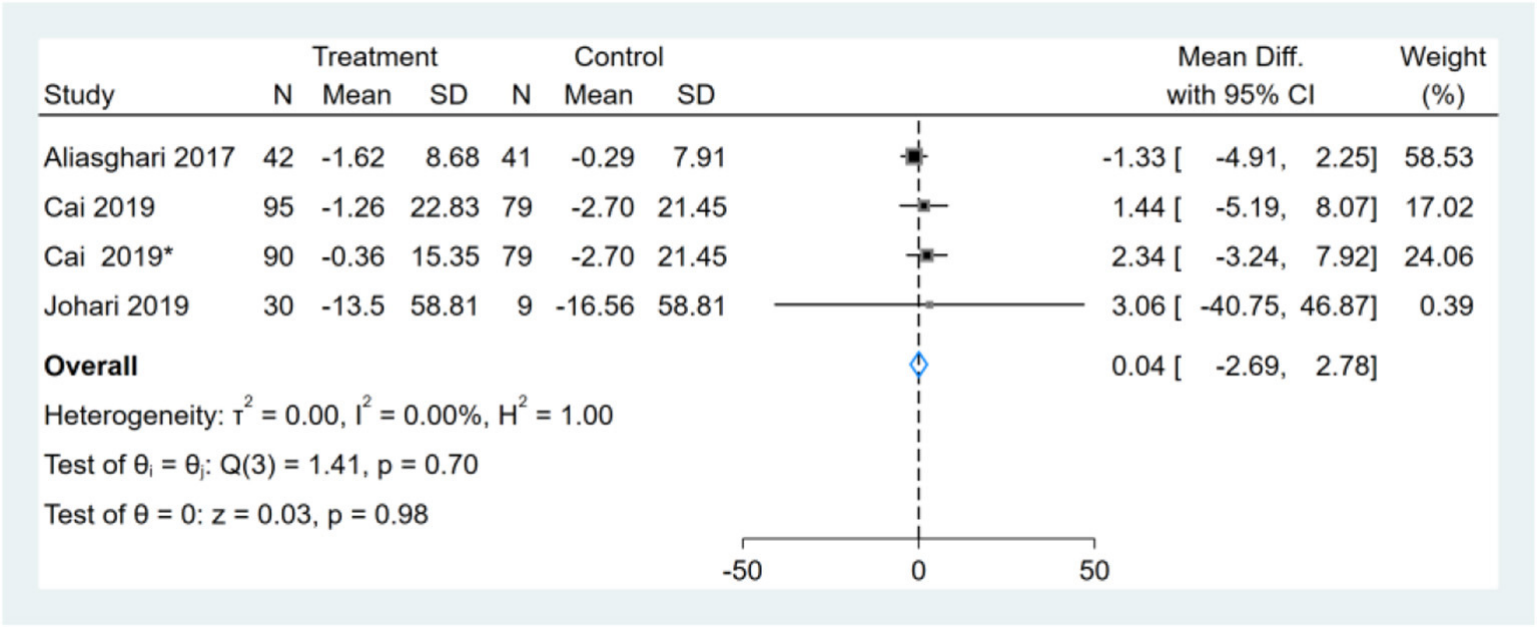
Forest plot analysis showing changes in fasting blood sugar/glucose (FBS/GLU). *This study has two fasting intervention groups.

Three studies reported FBS/GLU results, one of them had two comparators, and two datasets were included (13). Therefore, a total of four FBS/GLU datasets were included in the analysis. Meta-analysis shows that the effect of the fasting intervention was not significant for FBS/GLU (Figure 5).

### Effect of Intermittent Fasting on Serum HOMA-IR

**Figure 6:**
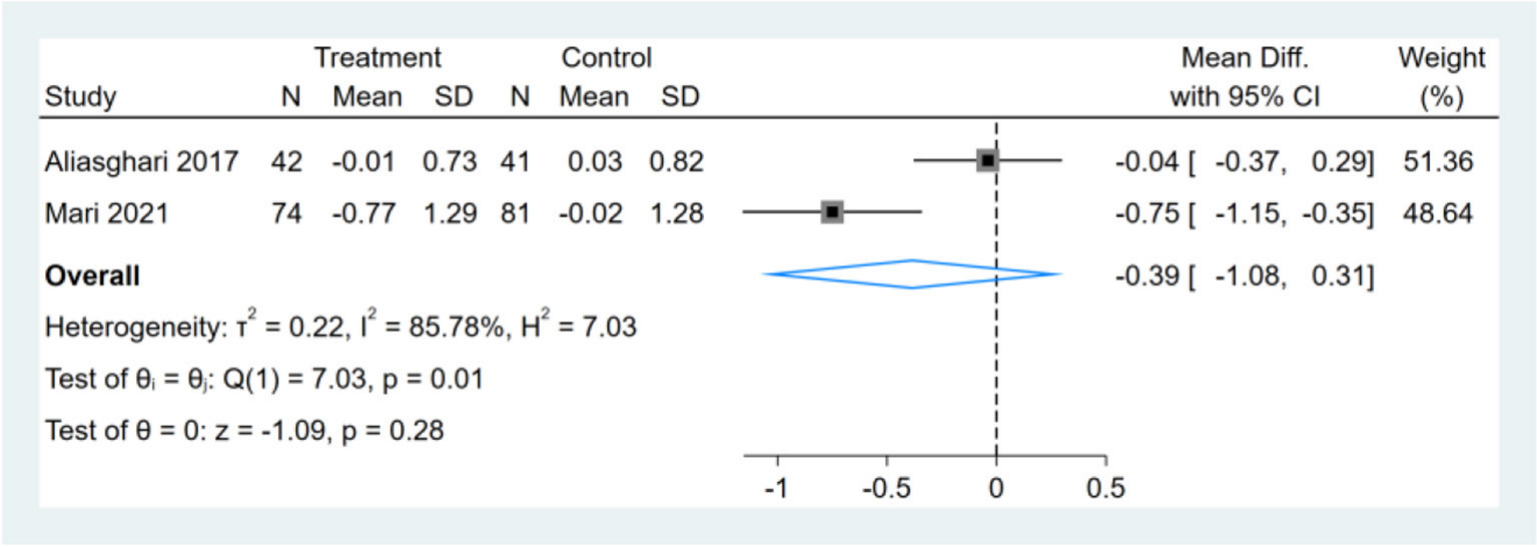
Forest plot analysis showing changes in homeostasis model assessment of insulin resistance (HOMA-IR).

Only two studies reported HOMA-IR results, and meta-analysis shows that the effect of the fasting intervention was not significant for HOMA-IR (Figure 6).

### Effect of Intermittent Fasting on Serum FINS

**Figure 7:**
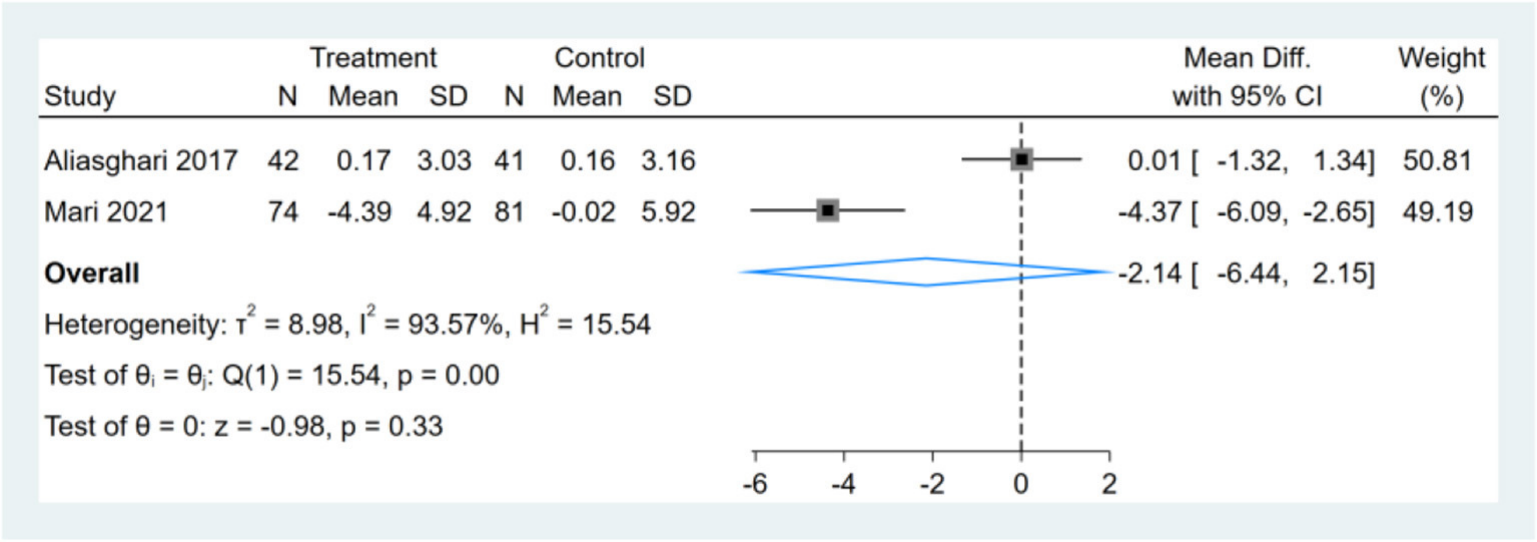
Forest plot analysis showing changes in fasting insulin (FINS).

Only two studies reported FINS results, and meta-analysis shows that the effect of the fasting intervention was not significant for FINS (Figure 7).

### Effect of Intermittent Fasting on Serum ALT

**Figure 8:**
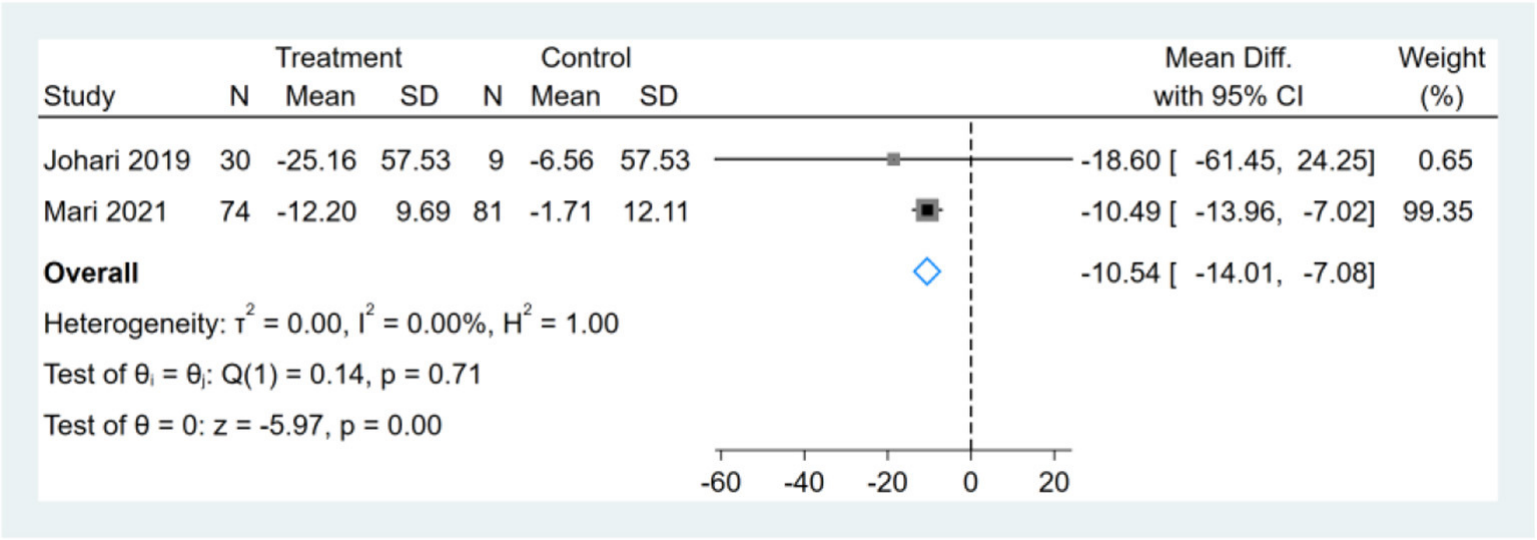
Forest plot analysis showing changes in alanine aminotransferase (ALT).

Three studies were included for analysis, which shows heterogeneity (*I*^2^ = 87%). We found the changing trend of ALT before and after the fasting reported in one study was significantly opposite to that in the previous study (17), and the heterogeneity was reduced after the deletion of this study (*I*^2^ = 0%). After the removal of the highly heterogeneous study, the remaining two studies were analyzed and showed that the effect of fasting on ALT was statistically significant (MD, −10.54, 95% CI from −14.01 to −7.08, *p* ≤ 0.00) (Figure 8).

### Effect of Intermittent Fasting on Serum AST

**Figure 9:**
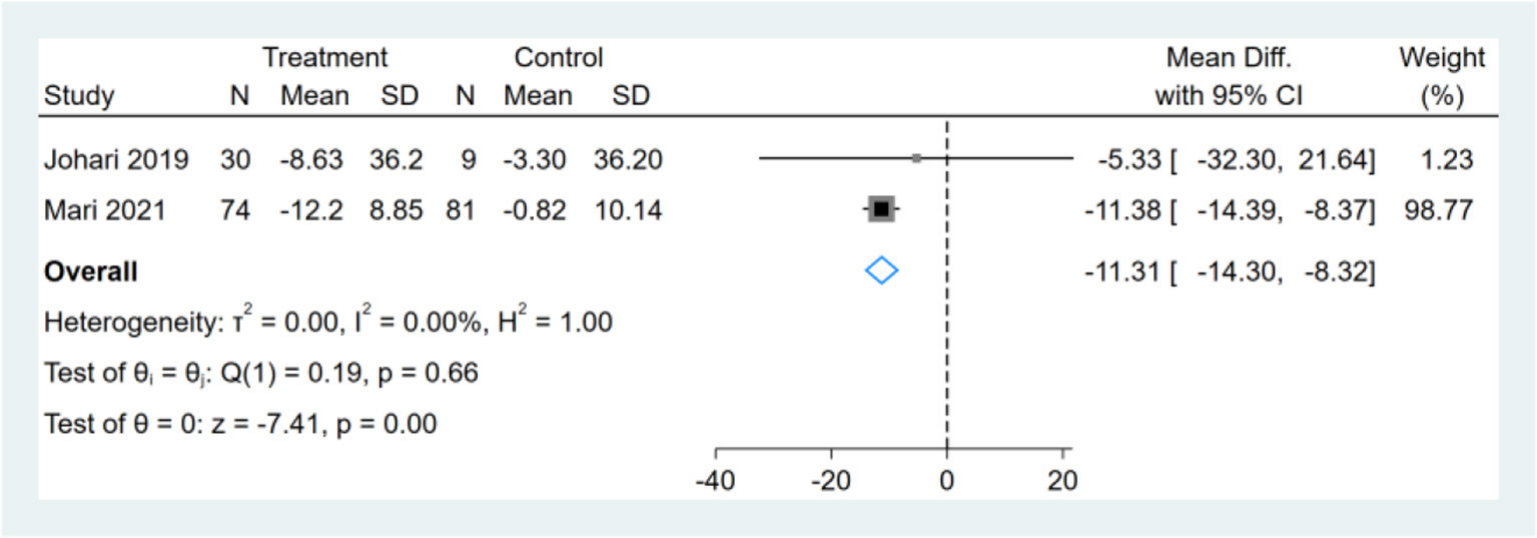
Forest plot analysis showing changes in aspartate transaminase (AST).

Only two studies reported AST results, and meta-analysis shows that the effect of fasting on AST was statistically significant (MD, −11.31, 95% CI from −14.30 to −8.32, *p* ≤ 0.00) (Figure 9).

### Effect of Intermittent Fasting on Liver Stiffness

**Figure 10:**
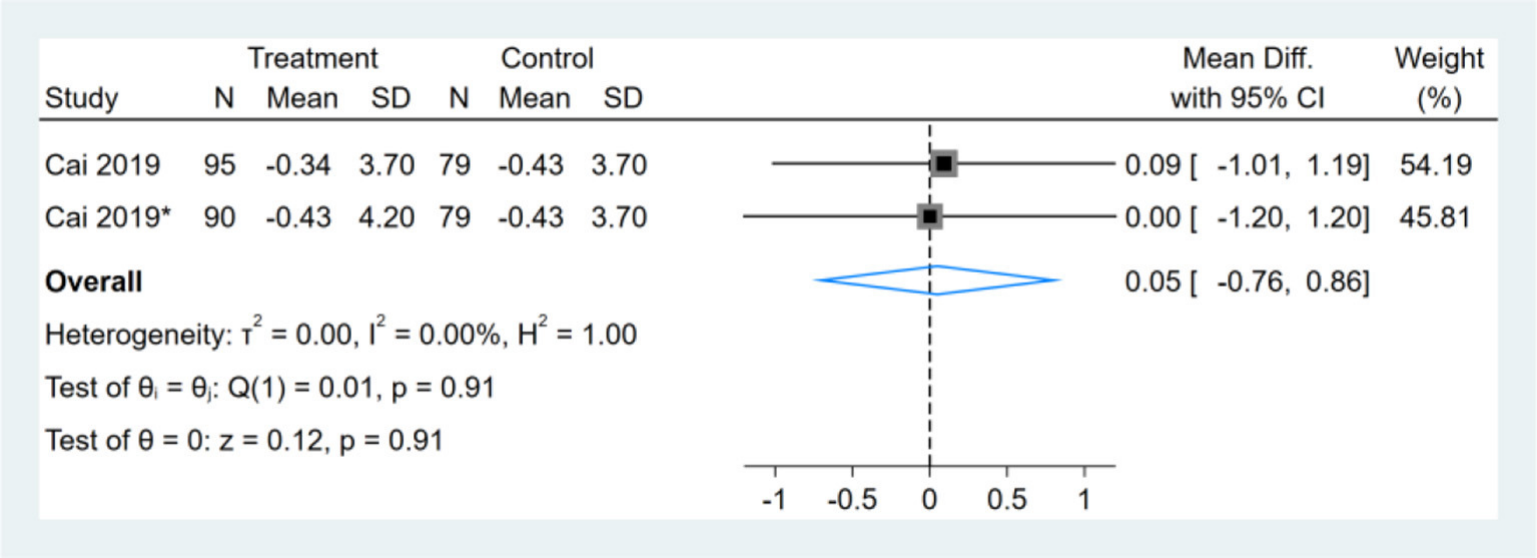
Forest plot analysis showing changes in liver stiffness. *This study has two fasting intervention groups.

Only one study reported liver stiffness results, which had two comparators, and two datasets were included. Therefore, a total of two liver stiffness datasets were included in the analysis. Meta-analysis shows that the effect of the fasting intervention was not significant for liver stiffness (Figure 10).

### Effect of Intermittent Fasting on Serum TG

**Figure 11:**
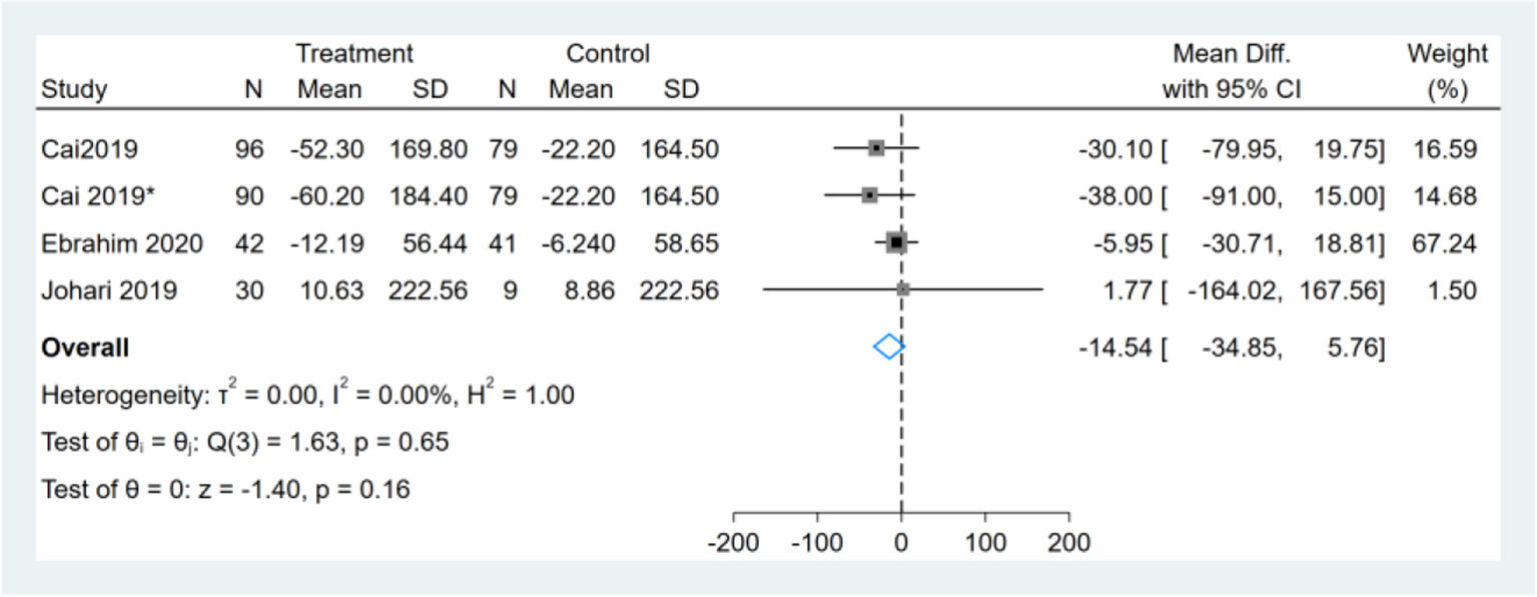
Forest plot analysis showing changes in triglyceride (TG). *This study has two fasting intervention groups.

Three studies reported TG results, one of them had two comparators, and two datasets were included (13). Therefore, a total of four TG datasets were included in the analysis. Meta-analysis shows that the effect of the fasting intervention was not significant for TG (Figure 11).

### Effect of Intermittent Fasting on Serum TC

**Figure 12:**
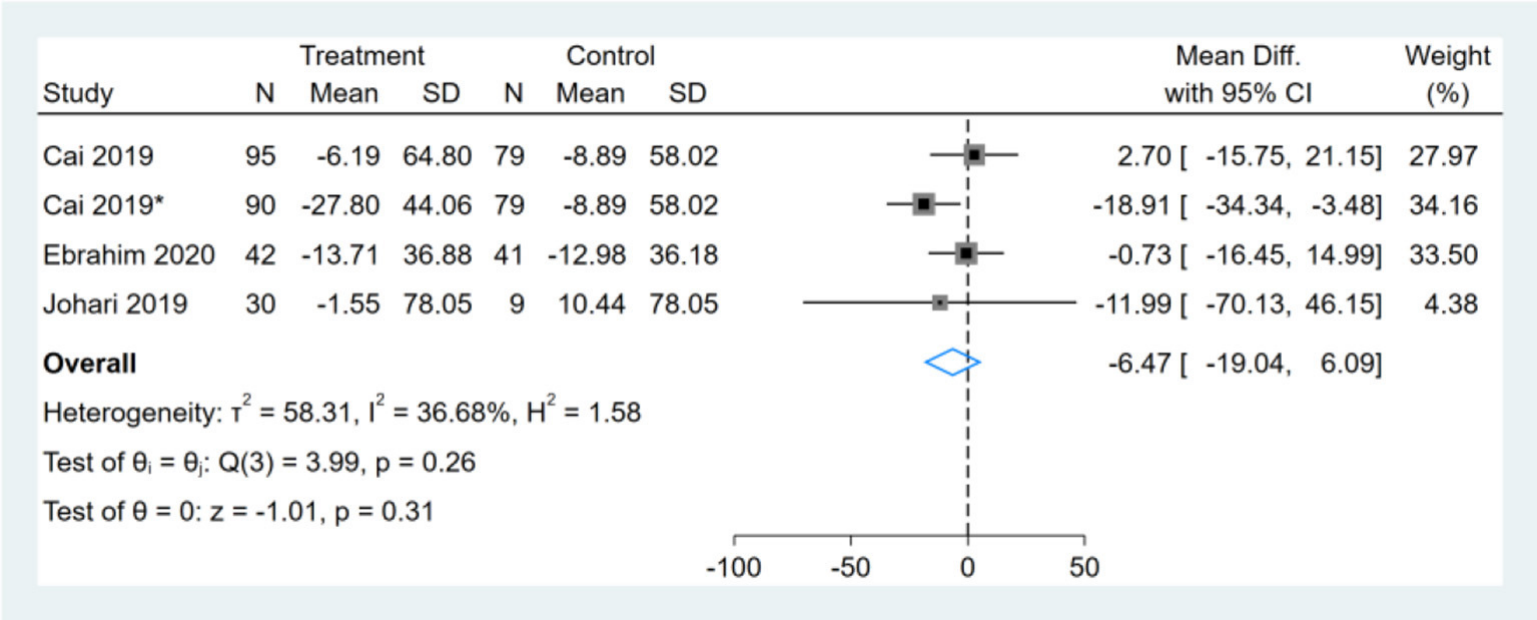
Forest plot analysis showing changes in total cholesterol (TC). *This study has two fasting intervention groups.

Three studies reported TC results, one of them had two comparators, and two datasets were included (13). Therefore, a total of four TC datasets were included in the analysis. Meta-analysis shows that the effect of the fasting intervention was not significant for TC (Figure 12).

### Effect of Intermittent Fasting on Serum LDL-C

**Figure 13:**
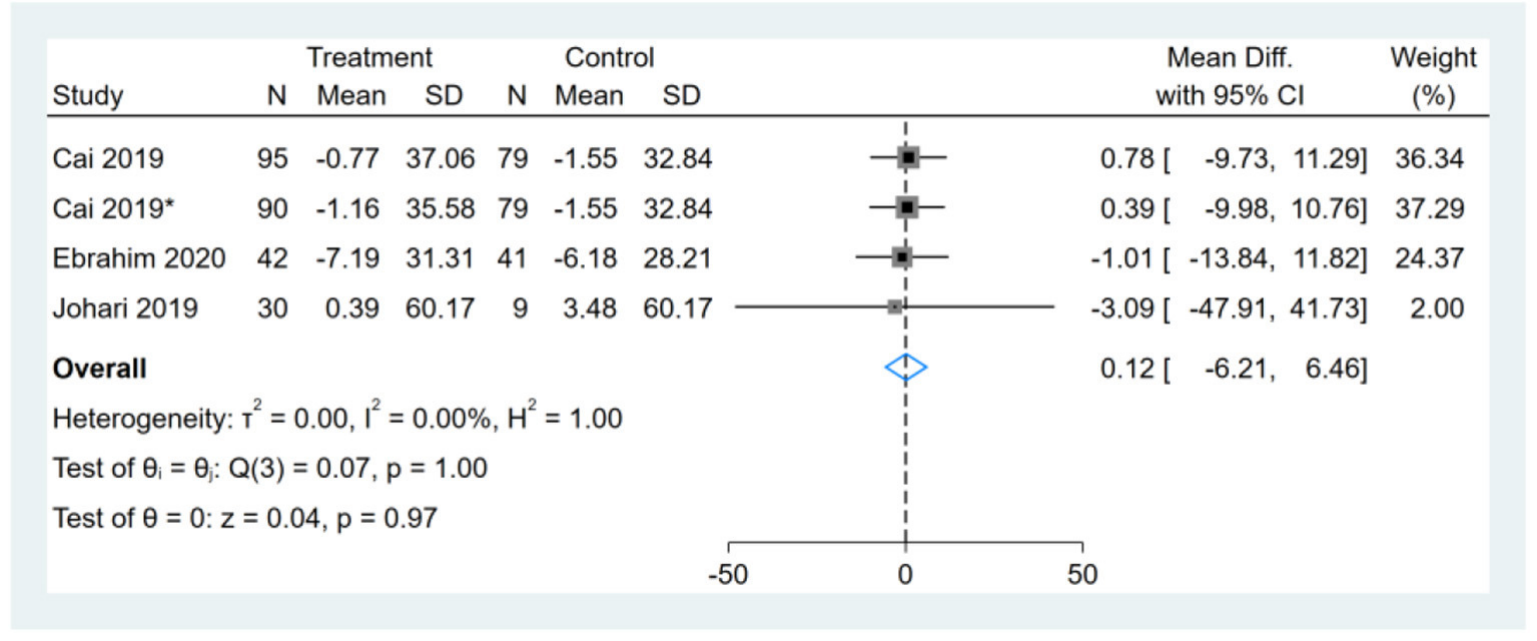
Forest plot analysis showing changes in low-density lipoprotein cholesterol (LDL-C). *This study has two fasting intervention groups.

Three studies reported LDL-C results, one of them had two comparators, and two datasets were included (13). Therefore, a total of four LDL-C datasets were included in the analysis. Meta-analysis shows that the effect of the fasting intervention was not significant for LDL-C (Figure 13).

### Effect of Intermittent Fasting on Serum HDL-C

**Figure 14:**
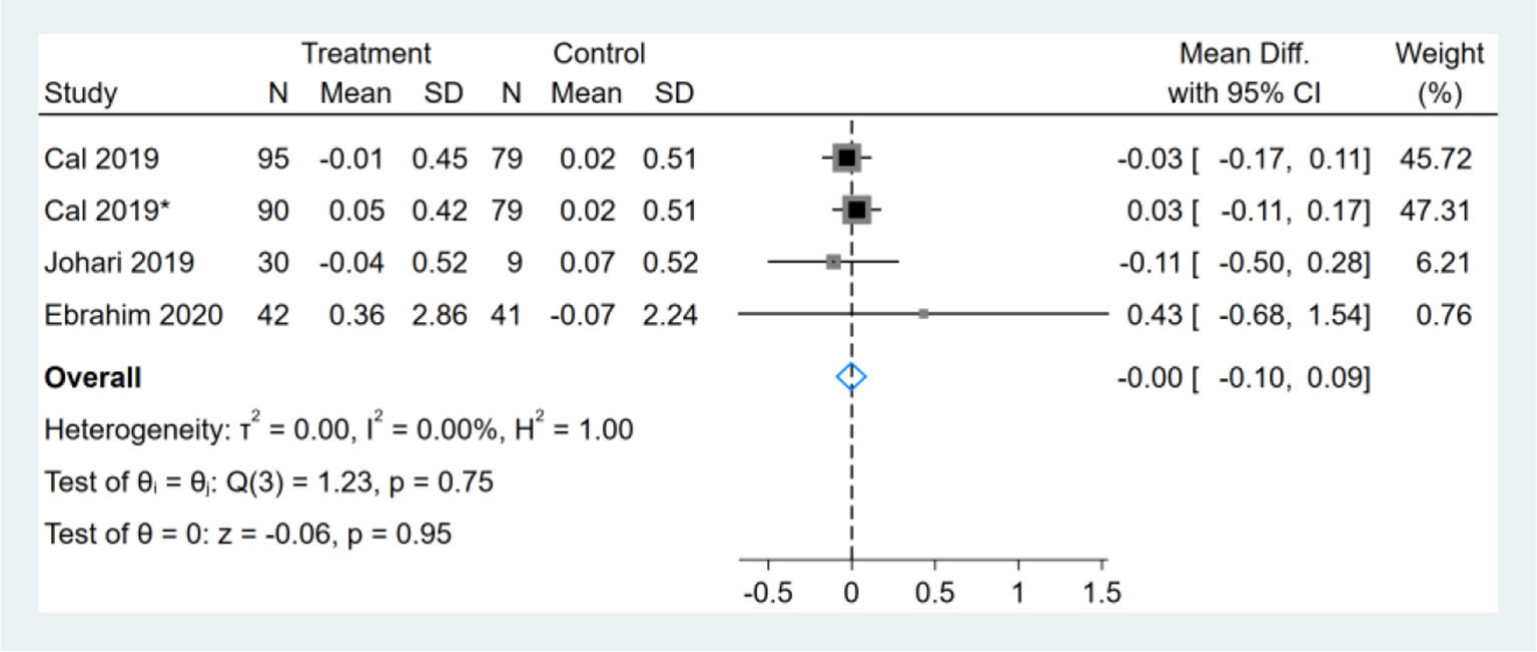
Forest plot analysis showing changes in high-density lipoprotein cholesterol (HDL-C). *This study has two fasting intervention groups.

Three studies reported HDL-C results, one of them had two comparators, and two datasets were included (13). Therefore, a total of four HDL-C datasets were included in the analysis. Meta-analysis shows that the effect of the fasting intervention was not significant for HDL-C (Figure 14).

### Subgroup Analysis

The pooled effect indicated that fasting significantly reduced the BW in subjects whose type of diet was modern intermittent fasting (−2.56; 95%CI: −4.19 −0.91) but not in the intermittent religious fasting (Table 2). BMI, WC, FBS/GLU, TG, TC, LDL-C, and HDL-C have no difference in the type of diet (Table 2). More investigations are needed as there were only a limited number of trials in each subgroup.

**Table 2:** Subgroup analyses of the effects of intermittent fasting on a fasting regimen.

**Type**	**Outcomes**	**No. of studies**	**No. of subjects**	**Meta-analysis**	**Heterogeneity**			
				**MD (95% CI)**	***Q* statistic**	***P* within group**	***I*^**2**^ (%)**	***P* between group**
Religious intermittent fasting	BW (kg)	2	144	−1.49 (−6.15, 3.18)	0.16	0.69	0	0.67
	BMI (kg/m^2^)	3	298	−1.09 (−2.54, 0.36)	3.21	0.21	30.74	0.38
	WC (cm)	1	83	−0.92 (−5.85, 4.01)	-	-	-	0.82
	FBS/GLU (mg/Dl)	1	83	−1.33 (−4.91, 2.25)	-	-	-	0.24
	TG (mmol/l)	1	83	−5.95 (−30.71, 18.81)	-	-	-	0.23
	TC (mmol/l)	1	83	−0.73 (−16.45, 14.99)	-	-	-	0.49
	LDL-C (mmol/l)	1	83	−1.01 (−13.84, 11.82)	-	-	-	0.84
	HDL-C (mmol/l)	1	83	0.43 (−0.68, 1.54)	-	-	-	0.44
Modern intermittent fasting	BW (kg)	3	382	**−2.56 (−4.19**, **−0.91)**	0.19	0.91	0	0.67
	BMI (kg/m^2^)	3	382	−0.41 (−0.87, 0.05)	0.57	0.75	0	0.38
	WC (cm)	2	343	−0.33 (−1.46, 0.75)	0.63	0.43	0	0.82
	FBS/GLU (mg/Dl)	3	382	1.98 (−2.27, 6.22)	1.41	0.7	0	0.24
	TG (mmol/l)	3	383	−32.18 (−67.65, 3.29)	0.21	0.9	0	0.23
	TC (mmol/l)	3	382	−9.15 (−27.15, 8.86)	3.99	0.26	36.68	0.49
	LDL-C (mmol/l)	3	382	0.49 (−6.80, 7.77)	0.07	1	0	0.84
	HDL-C (mmol/l)	3	382	−0.01 (−0.10, 0.09)	1.23	0.75	0	0.44

*Significant results are in bold (P < 0.05)*.

### Risk of Bias

**Figure 15:**
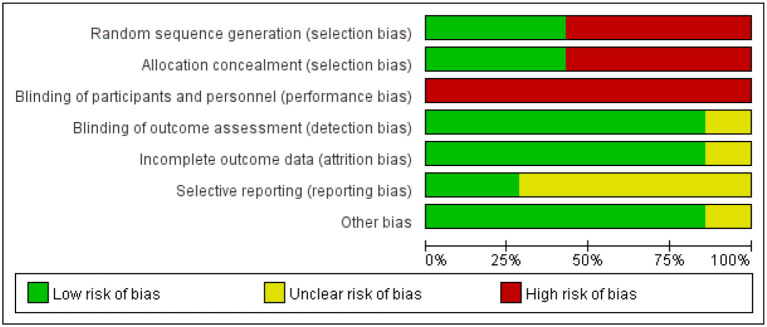
Risk of bias graph identified in the studies included in the meta-analysis.

**Figure 16:**
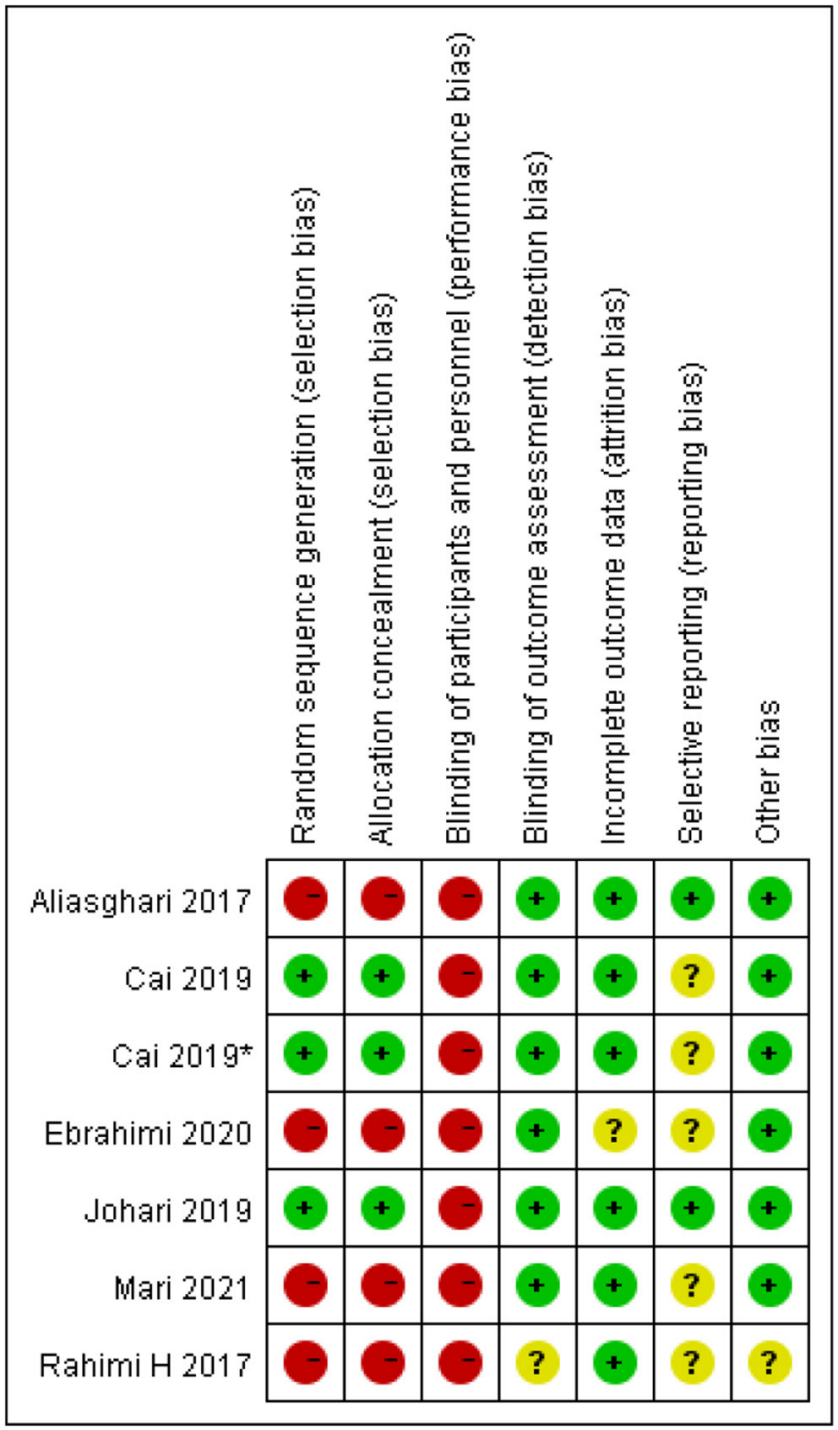
Risk of bias summary identified in the studies included in the meta-analysis. *This study has two fasting intervention groups.

The risk of bias assessment is shown in Figures 15, 16. We assess all the six studies according to the guidelines in the Cochrane Handbook for Systematic Reviews of Interventions. The blinding of the participants was limited because of the characteristics of the intervention. Consequently, we assess all the six studies as high risk of performance bias. Four studies did neither evaluate the generation of randomization sequence nor adequately conceal allocations.

### Publication Bias

Based on the visual inspection of funnel plots, there may be publication deviations (Figures 17–29). However, the number of included trials was small in each analysis. Thus, further investigations are needed.

**Figure 17:**
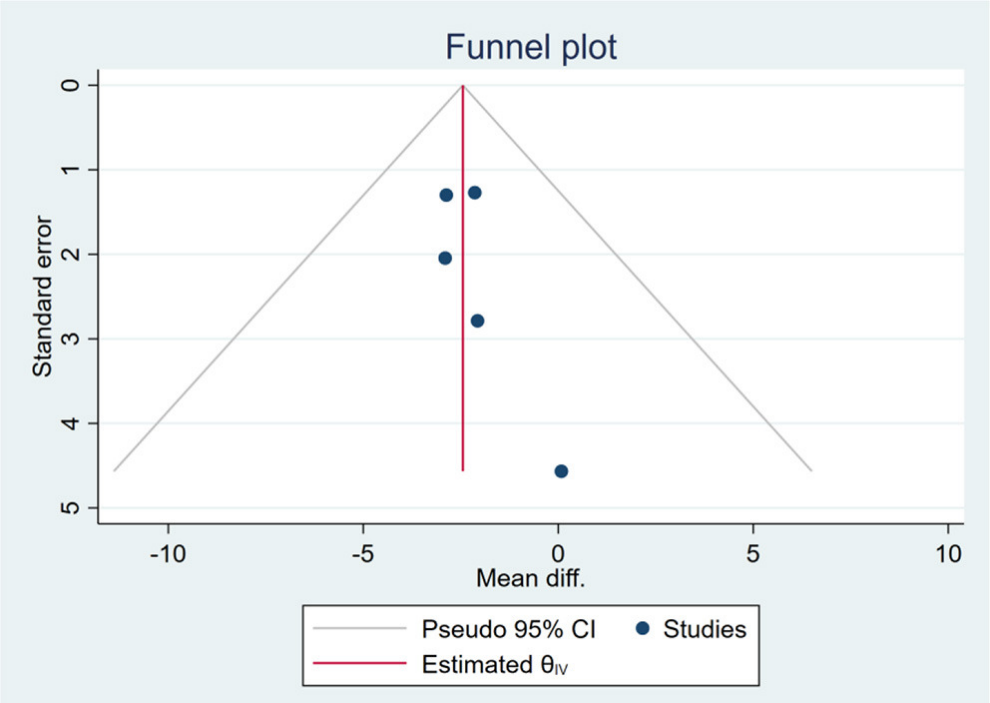
Funnel plots of the effect of body weight (BW).

**Figure 18:**
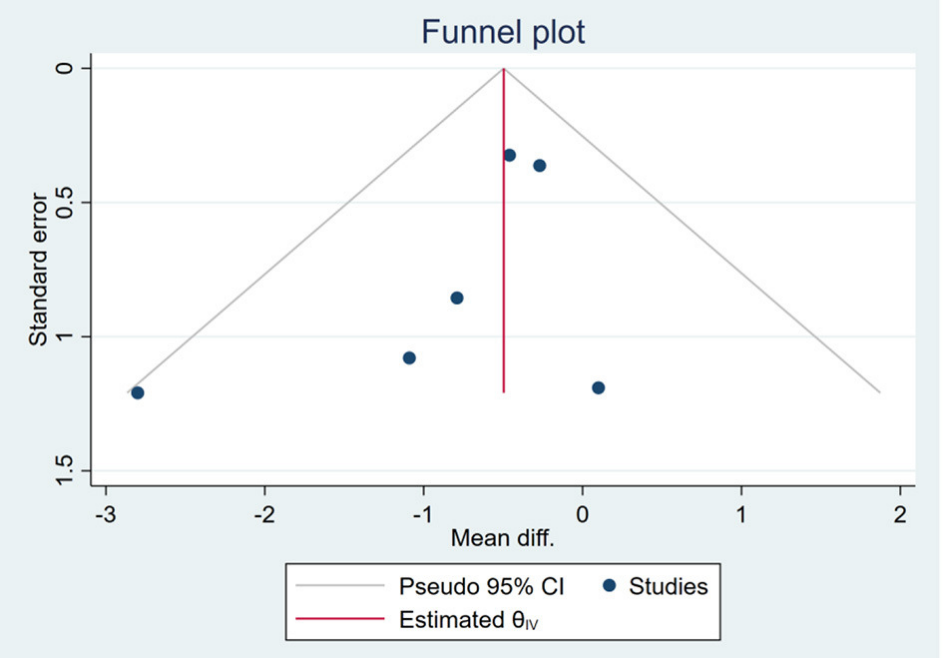
Funnel plots of the effect of body mass index (BMI).

**Figure 19:**
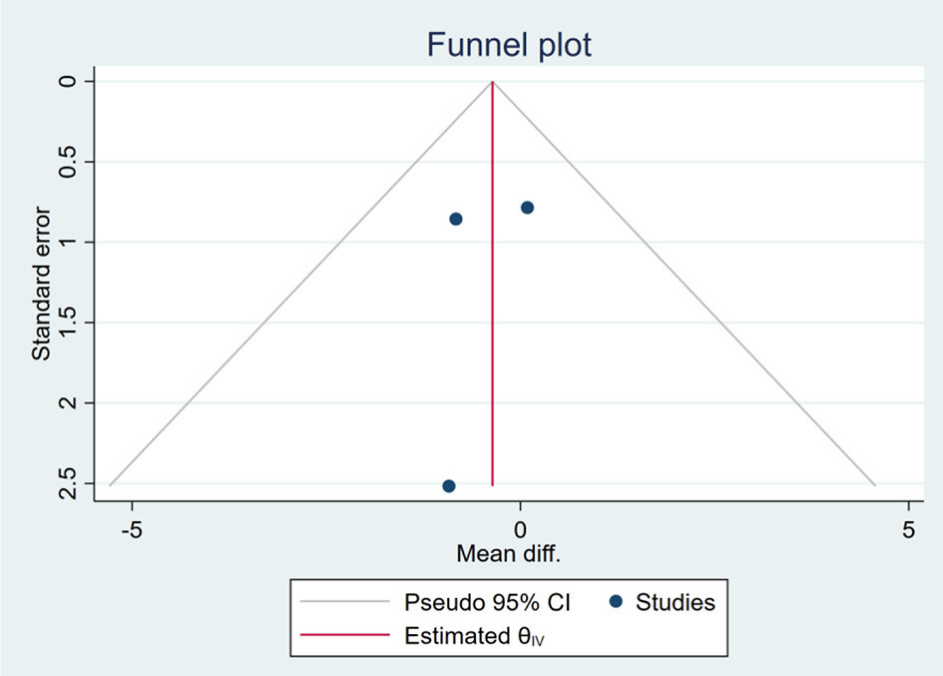
Funnel plots of the effect of waist circumference (WC).

**Figure 20:**
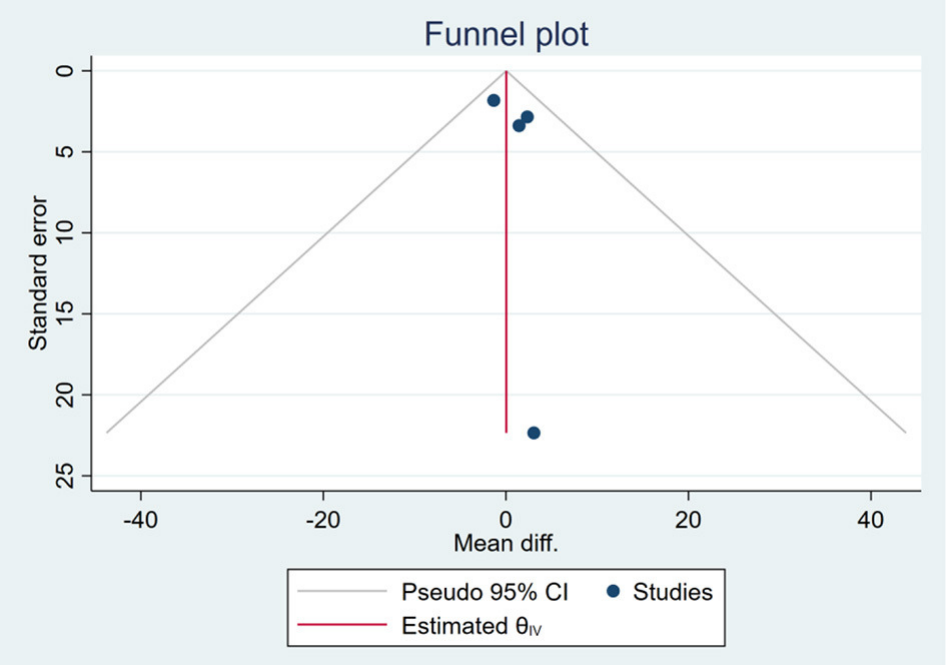
Funnel plots of the effect of FBS/GLU.

**Figure 21:**
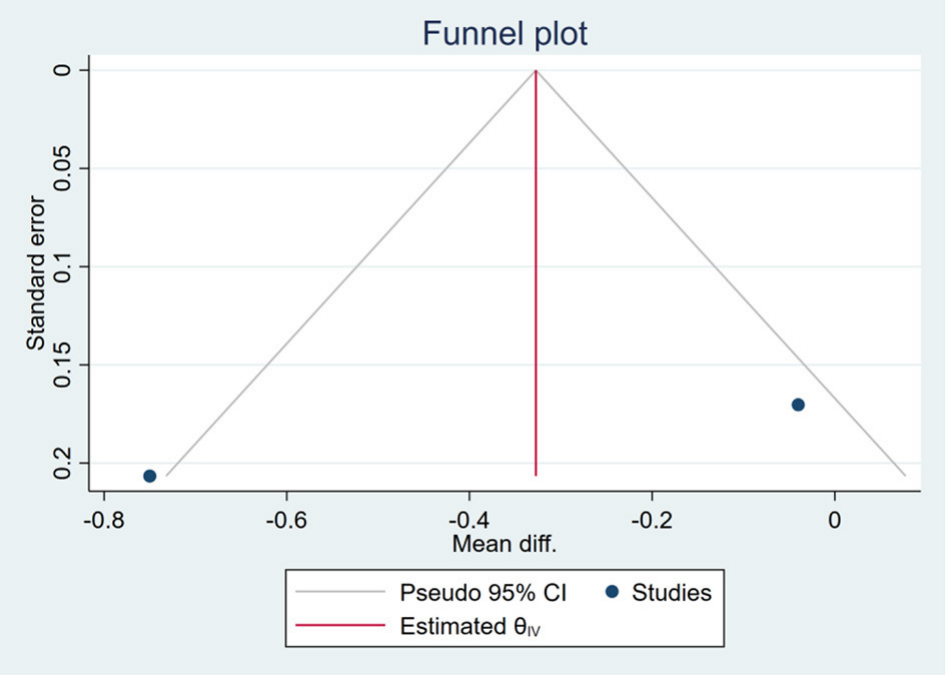
Funnel plots of the effect of HOMA-IR.

**Figure 22:**
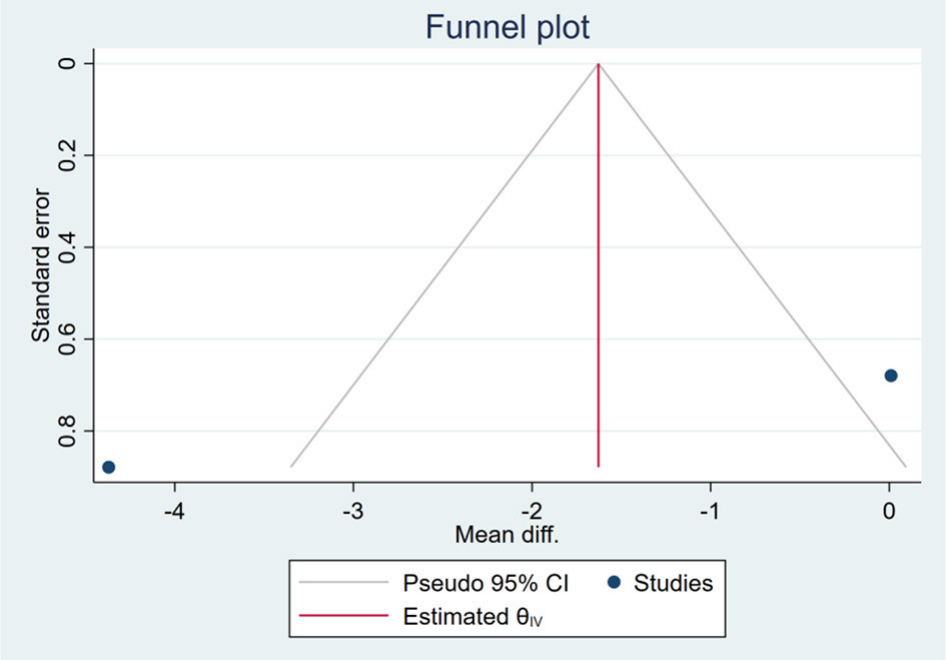
Funnel plots of the effect of FINS.

**Figure 23:**
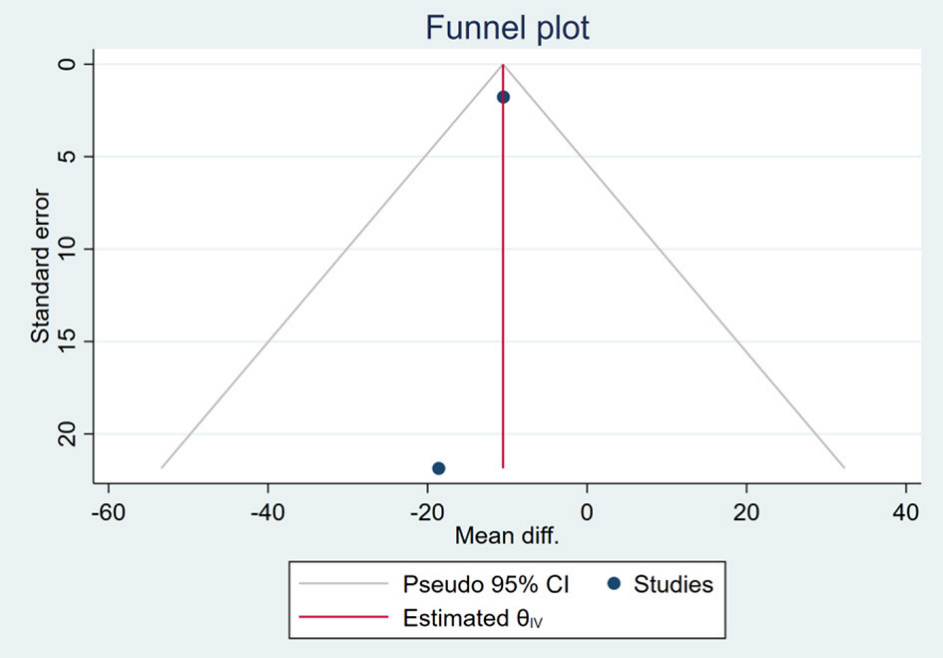
Funnel plots of the effect of ALT.

**Figure 24:**
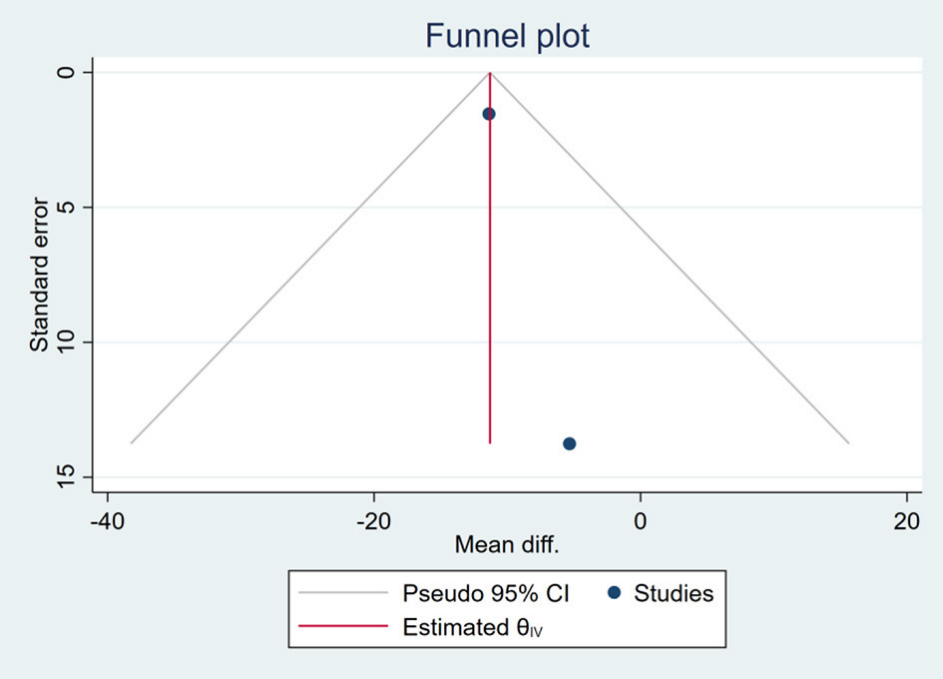
Funnel plots of the effect of AST.

**Figure 25:**
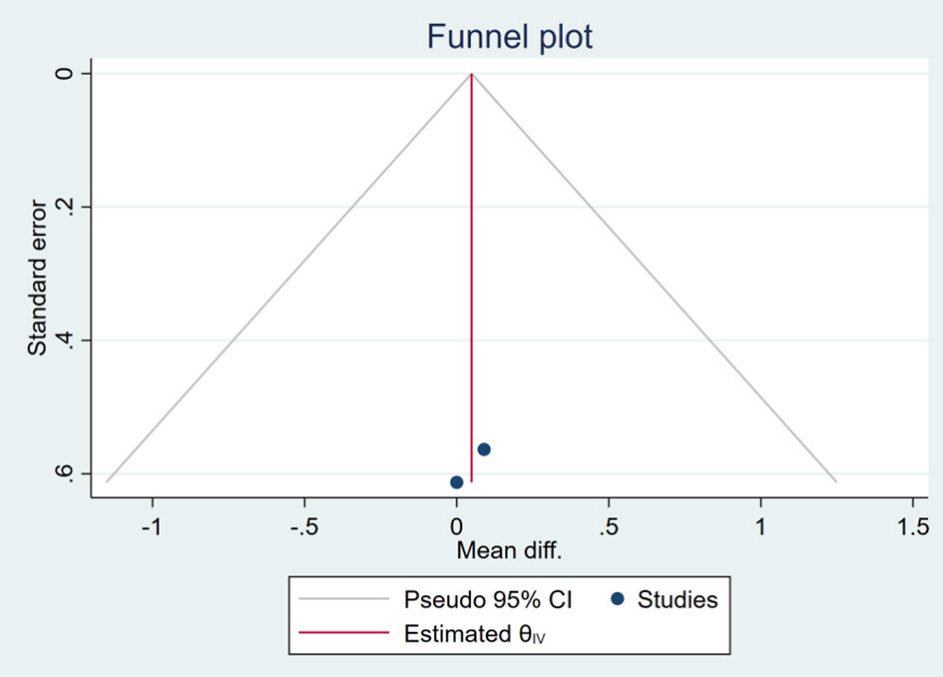
Funnel plots of the effect of liver stiffness.

**Figure 26:**
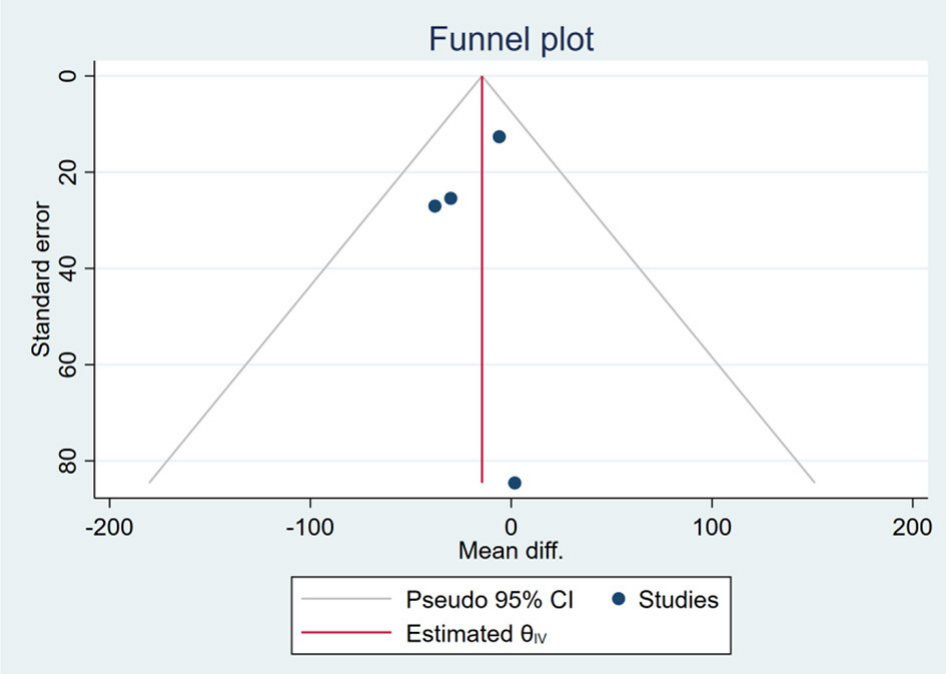
Funnel plots of the effect of TG.

**Figure 27:**
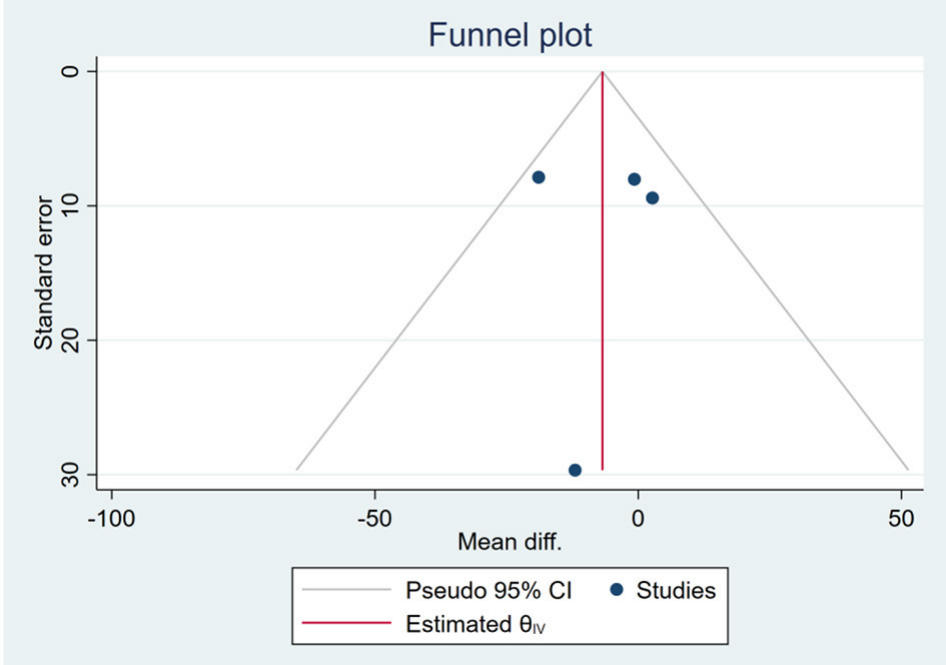
Funnel plots of the effect of TC.

**Figure 28:**
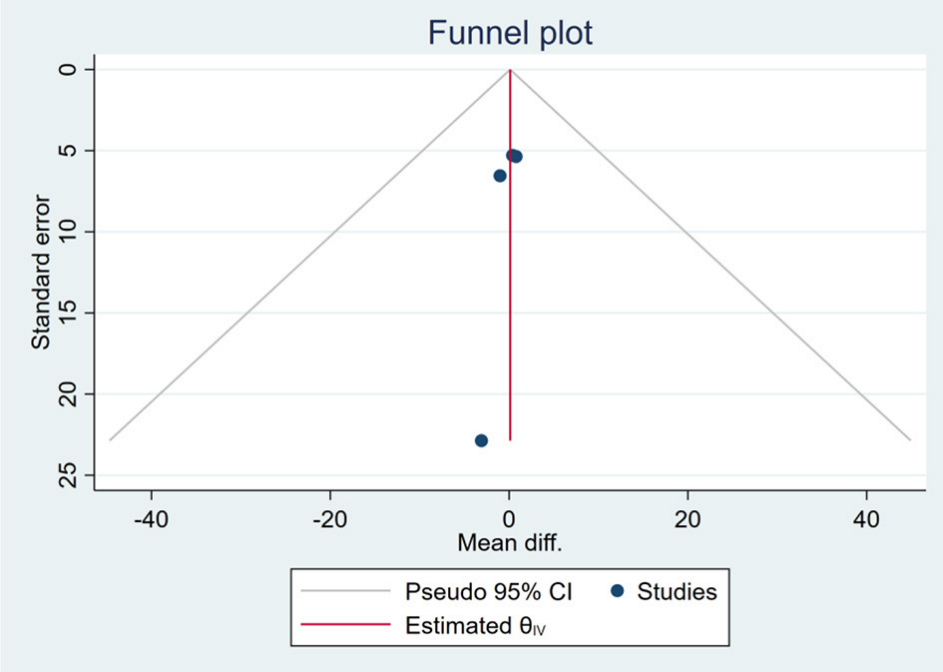
Funnel plots of the effect of LDL-C.

**Figure 29:**
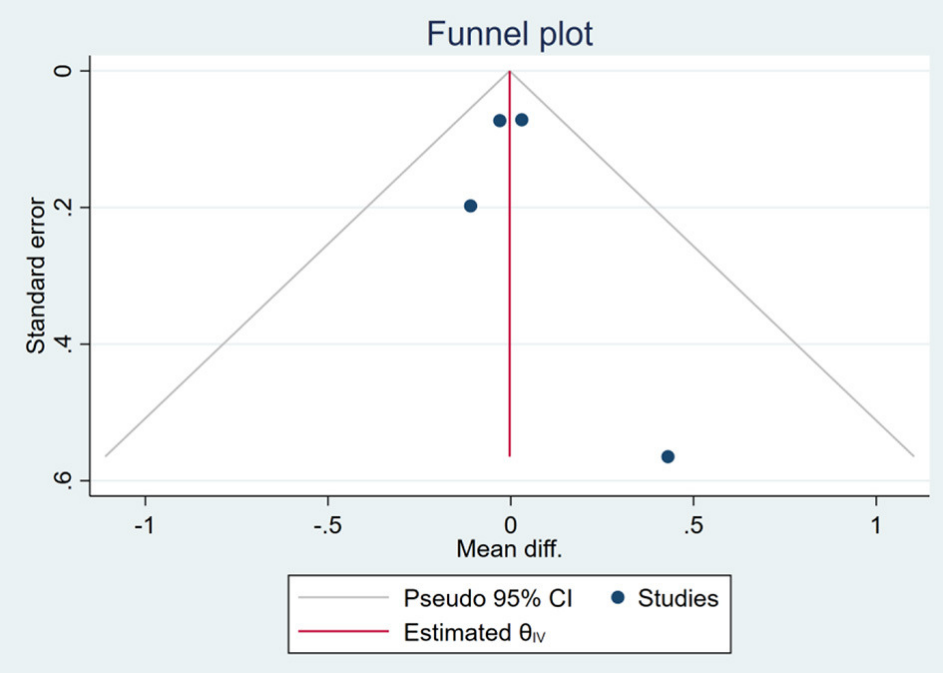
Funnel plots of the effect of HDL-C.

## Discussion

Obesity results from excess calories are a significant risk factor for developing insulin resistance (IR), a critical factor in the etiology of metabolic syndrome and type 2 diabetes mellitus (20, 21). In turn, IR promotes the development of NAFLD in a subset of patients that could evolve to a more severe form, i.e., NASH. Unfortunately, science is far from thoroughly clarifying the genesis of NAFLD and suggesting appropriate therapy to reduce or eliminate them drastically (22). At present, there are no approved pharmacological therapies for NAFLD. Many guidelines advocate recommendations focusing on controlling risk factors and lifestyle changes that include dietary and physical activities (15). Dietary restriction increases the endogenous synthesis of fatty acids. Dietary and endogenously synthesized fatty acids contribute to the whole-body fatty acid pool (22, 23). Intermittent fasting is the established diet paradigm that extends life span and health span across species (21, 24).

Diet and exercise are among the most commonly used ones but complicated to maintain due to the ability of each organism to balance the reduced intake of calories with a decrease in resting metabolic rate, thus frustrating the beneficial effect (25). Therefore, based on the medical history and preferences of patients, appropriate individualized diet and exercise prescriptions should be developed. This is best approached with a healthcare team including a physician, registered dietitian, and exercise physiologist. Through this slow and thoughtful process of cycles of weight loss and weight maintenance, it is thought that patients will be able to prevent the more debilitating cycles of rapid weight loss, short-term reductions in metabolic rate, and rapid weight gain (25).

This study is the first time to evaluate the effectiveness of intermittent fasting on weight loss and improvement of liver function-related parameters in patients with NAFLD. On the basis of this meta-analysis, we confirmed an improvement in ALT and AST through fasting compared with a non-fasting control group. This seems to be related to a decrease in BW and a reduction in BMI. Earlier meta-analyses have shown similar results that intermittent fasting was more effective than the control for BMI and BW (9, 10).

Studies in rodents have demonstrated that TRF improves metabolic profiles and reduces the risk of obesity and its related conditions (26, 27). Similarly, all six studies in our meta-analysis that directly compared the fasted group with the non-fasted group showed that fasting was not only beneficial for weight and BMI reduction but also for ALT and AST reduction, with a statistical significance. By pooling the data, we provide clear evidence that fasting can reduce BW, BMI, ALT, and AST. None of the six included studies were confounded by exercise or other interventions. Therefore, intermittent fasting has an independent and significant benefit on weight loss and improvement of liver function in patients with NAFLD. Recent research studies reported similar results that short-term, isocaloric TRF would diminish the severity of NAFLD and improve metabolic parameters associated with liver function in short-term diet-restricted fasted mice (28). The beneficial effects of fasting are often regarded as driven by reductions in BW and/or body fat (29). In addition, the mechanism for the direct hepatic benefit of fasting is considered to be related to its effect on circadian biology, gut microbiome, and modifiable lifestyle behaviors (26, 30–33).

We failed to observe the benefits of fasting on metabolism-related parameters such as TG, TC, LDL-C, HDL-C, FBS/GLU, HOMA-IR, and FINS. Among the included studies, four studies reported improvements in lipid metabolism indicators, such as TG, TC, LDL-C, and HDL-C. In contrast, the above indicators did not show significant differences during statistical analysis by STATA. This may be related to the fact that there are few available literature studies with high heterogeneity among them. Thus, the effect of fasting on lipid metabolism needs to be further confirmed by more clinical RCTs.

This meta-analysis provides evidence for the effect of intermittent fasting on weight reduction and improvement of liver function. However, few included studies and the low sample size would affect and limit the meta-analysis results. Four of the six included studies were religion-related fasting, and only two were RCTs, which is also an important reason for heterogeneity. More studies on large-scale randomized controlled intervention are needed to better inform clinical recommendations for fasting interventions in NAFLD. Long-term control and maintenance of weight are more important, while follow-up support is lacking in the six studies included this time. In addition, safety is essential for fasting interventions but is severely lacking.

## Conclusions

Intermittent fasting is beneficial for weight management and liver enzyme improvement, but long-term feasibility and safety of intermittent fasting should be conducted in further studies.

## Data Availability Statement

The original contributions presented in the study are included in the article/Supplementary Material, further inquiries can be directed to the corresponding author/s.

## Author Contributions

CY and JL contributed to conception and design of the study and wrote the first draft of the manuscript. CY and MH organized the database. CY performed the statistical analysis. ZHL, YLX, HBP, PY, XYZ, SJY, and YW wrote sections of the manuscript. All authors contributed to manuscript revision, read, and approved the submitted version.

## Conflict of Interest

The authors declare that the research was conducted in the absence of any commercial or financial relationships that could be construed as a potential conflict of interest.
